# Electronic-Based Patient-Reported Outcomes: Willingness, Needs, and Barriers in Adjuvant and Metastatic Breast Cancer Patients

**DOI:** 10.2196/cancer.6996

**Published:** 2017-08-07

**Authors:** Andreas D Hartkopf, Joachim Graf, Elisabeth Simoes, Lucia Keilmann, Nina Sickenberger, Paul Gass, Diethelm Wallwiener, Lina Matthies, Florin-Andrei Taran, Michael P Lux, Stephanie Wallwiener, Eric Belleville, Christof Sohn, Peter A Fasching, Andreas Schneeweiss, Sara Y Brucker, Markus Wallwiener

**Affiliations:** ^1^ Department of Women’s Health University Hospital Tuebingen Tübingen Germany; ^2^ Research Institute for Women's Health Department of Women’s Health University Hospital Tuebingen Tübingen Germany; ^3^ Hospital for General Obstetrics and Gynecology National Cancer Center University Hospital Heidelberg Heidelberg Germany; ^4^ Department of Gynecology and Obstetrics University Hospital Erlangen University Breast Center Franconia, Comprehensive Cancer Center Erlangen Erlangen Germany; ^5^ Clin-Sol Ltd. Würzburg Germany

**Keywords:** breast cancer, patient-reported outcome measures, electronic patient- reported outcome, technical skills, willingness to use, needs and barriers

## Abstract

**Background:**

Patient-reported outcomes (PROs) play an increasingly important role as an adjunct to clinical outcome parameters in measuring health-related quality of life (HRQoL). In fact, PROs are already the accepted gold standard for collecting data about patients’ subjective perception of their own state of health. Currently, paper-based surveys of PRO still predominate; however, knowledge regarding the feasibility of and barriers to electronic-based PRO (ePRO) acceptance remains limited.

**Objective:**

The objective of this trial was to analyze the willingness, specific needs, and barriers of adjuvant breast cancer (aBC) and metastatic breast cancer (mBC) patients in nonexposed (no exposure to electronic assessment) and exposed (after exposure to electronic assessment decision, whether a tablet-based questionnaire is favored) settings before implementing digital ePRO assessment in relation to health status. We also investigated whether providing support can increase the patients’ willingness to participate in such programs.

**Methods:**

The nonexposed patients only answered a paper-based questionnaire, whereas the exposed patients filled out both paper- and tablet-based questionnaires. The assessment comprised socioeconomic variables, HRQoL, preexisting technical skills, general attitude toward electronic-based surveys, and potential barriers in relation to health status. Furthermore, nonexposed patients were asked about the existing need for technological support structures. In the course of data evaluation, we performed a frequency analysis as well as chi-square tests and Wilcoxon signed-rank tests. Subsequently, relative risks analysis, univariate categorical regression (CATREG), and mediation analyses (Hayes’ bias-corrected bootstrap) were performed.

**Results:**

A total of 202 female breast cancer patients completed the PRO assessment (nonexposed group: n=96 patients; exposed group: n=106 patients). Self-reported technical skills were higher in exposed patients (2.79 vs 2.33, *P* ≤.001). Significant differences were found in relation to willingness to use ePRO (92.3% in the exposed group vs 59% in the nonexposed group; *P*=.001). Multiple barriers were identified, and most of them showed statistically significant differences in favor of the exposed patients (ie, data security [13% in the exposed patients vs 30% in the nonexposed patients; *P*=.003] and no prior technology usage [5% in the exposed group vs 15% in the nonexposed group; *P*=.02]), whereas the differences in disease burden (somatic dimension: 4% in the exposed group vs 9% in the nonexposed group; *P*=.13) showed no significance. In nonexposed patients, requests for support services were identified, which could increase their ePRO willingness.

**Conclusions:**

Significant barriers in relation to HRQoL, cancer-related restrictions, and especially the setting of the survey were identified in this trial. Thus, it is necessary to address and eliminate these barriers to ensure data accuracy and reliability for future ePRO assessments. Exposure seems to be a potential option to increase willingness to use ePRO and to reduce barriers.

## Introduction

### Patient-Reported Outcomes in Breast Cancer Patients

Current advances in immuno-oncology and various target treatment combinations provide promising results such as long-term survival in cancer patients. However, treatment environments are challenging with regard to balancing clinical outcome and monitoring of quality of life, for example, in breast cancer patients [1,2]. Hence, patient-reported outcomes (PROs) play an increasingly important role as an adjunct to clinical outcomes in clinical practice [3]. A PRO is defined as “any report of the status of a patient’s health condition that comes directly from the patient, without interpretation of the patient’s response by a clinician or anyone else” [4]. PROs comprise various aspects of the subjectively perceived state of health from the patient’s point of view, such as the health-related quality of life (HRQoL) [3-9].

### Novel Assessment of HRQoL and Adverse Events in Clinical Routine

PROs are assumed to be versatile and heterogeneous because they implicate many health conditions such as HRQoL, symptom severity (eg, using a pain scale), physical mobility, degree of psychological stress, disease-related impairment in daily routine, patient satisfaction, and drug adherence [3-13]. HRQoL is an important tool in clinical routine that comprises physical, emotional, mental, social, and behavioral components in terms of the patient’s well-being and functioning from the patient’s subjective perspective [12-15]. Furthermore, PROs reflect treatment success in a patient-centered manner [3-5,15-18]. Thus, PROs should be used to measure the *effectiveness* of new interventions to complement the results of *efficacy studies*, which only evaluate the success of therapeutic interventions in a clinical trial [4,18]. In the case of oncology patients, the patients’ subjective perception of their own state of health is considered an important indication of the efficacy and safety of a specific therapy [19-25]. For example, in patients with metastatic breast cancer, PROs are a relevant source of information indicating whether the primary treatment aim of prolonging life with a more reasonable HRQoL is achieved [24,26-29]. The relevance of validated PRO questionnaires (eg, the European Organization for Research and Treatment of Cancer Quality of Life questionnaire-Core 30 item [EORTC QLQ-C30]) has been confirmed in several studies, in which patients with chronic diseases assessed their quality of life as being significantly worse, as compared with the clinical assessment [27-31]. Due to its high practicability and validity, EORTC QLQ-C30 is one of the most commonly used questionnaires for measuring PRO in patients with breast cancer.

### Electronic Monitoring of PRO on the Rise

Validated PROs for measuring cancer-specific HRQoL (eg, EORTC QLQ-C30 and Functional Assessment of Cancer Therapy-General [FACT-G]) are already the accepted gold standard for data collection for closely related variables such as HRQoL, satisfaction with care, and drug adherence [32-34]. Currently, paper-based surveys of PRO still predominate in clinical routine, especially because there is a lack of validated electronic-based PRO (ePRO) measurement instruments pertaining to various oncological conditions [35]. There is growing demand for information and communication on behalf of patients and increasing integration of information technology in health care, which is why data on patient-relevant end points has increasingly been collected electronically in recent years. Thus, the potential of electronic health (eHealth) solutions in health care research is becoming increasingly apparent [36,37]. The benefits of digital data capture include real-time data capture, screening for deterioration of adverse events (AEs), potential cost-effectiveness for health centers, and therefore, a potential for longitudinal symptom assessment [38,39]. Long-term digital AE monitoring also seems feasible. Nevertheless, knowledge regarding patient acceptance, feasibility, and barriers remains limited, especially in relation to health status and socioeconomic aspects [40-45]. Previously collected data regarding barriers showed that older metastatic breast cancer patients with a higher disease burden may be less inclined to complete ePRO questionnaires (eg, by using tablet devices) [46]. The technical experience and skills of the patient population also have a significant impact on the adoption and adherence rates. Patients who participated in Web-based symptom monitoring showed both a 16% higher improvement in HRQoL and a 6% higher 12-month overall survival, were 7% less frequently admitted to the emergency room [38], and were more willing to use ePRO [46]. To date, little research has been conducted on whether the willingness to use eHealth applications increases when patients are exposed to it. No studies could be identified which focalize on whether the use of ePRO can be increased or potential barriers alleviated by exposure. However, studies from geriatrics indicate that reservations of elderly patients can be deferred to eHealth applications when faced directly with them [47-49]. It is also unclear to what extent sociodemographic variables influence exposure. For reliable and valid measurement of ePRO, it is relevant to identify all the variables that influence patients’ response behavior.

### Aims

The main aim of this study was to analyze the willingness, specific needs, and barriers of adjuvant breast cancer (aBC) and metastatic breast cancer (mBC) patients before implementing digital ePRO assessment in relation to health status (HRQoL and therapy setting [aBC vs mBC]). We also investigated whether providing support can increase their willingness to participate in such programs. We analyzed potential differences in the willingness of aBC and mBC patients in relation to the survey setting (nonexposed vs exposed survey). The main aim of the study was to analyze the influence of an ePRO tool on the patients’ willingness to participate. Second, possible hurdles for ePRO that determine nonresponse rates should be identified in breast cancer patients. With the long-term goal being to use ePRO exclusively, appropriate barriers must be identified. This trial evaluated the patients’ general acceptance and practicability of ePRO in aBC and mBC subgroups. The goal was to analyze whether there was coherence between the health status (aBC vs mBC) and the willingness/frequency of barriers and between the survey setting (nonexposed vs exposed survey) and the willingness/frequency of barriers. To achieve the aims, aBC and mBC patients with and without ePRO exposure were asked to fill out questionnaires about their sociodemographic indications, technical skills, HRQoL, willingness to use, and potential barriers.

## Methods

### Sample and Study Design

From July 2015 to May 2016, paper-based PRO questionnaires were completed by female aBC and mBC patients treated consecutively at the Department of Women’s Health in Tuebingen, Germany, and the National Cancer Center in Heidelberg, Germany. To analyze the dependency of identified barriers regarding health status in aBC and mBC patients, we compared nonexposed and exposed patients. The patients were recruited from two different studies: 106 exposed patients were recruited from electronic-based Patient-Reported Outcomes and Compliance Analysis (ePROCOM) and 96 nonexposed patients from another study [46]. All female breast cancer patients aged more than 18 years who either had metastasis or were undergoing adjuvant treatment, who additionally had sufficient knowledge of German to answer the questionnaire, and who declared their consent to fill out the questionnaires during an outpatient visit to the hospital under the supervision of an attending physician were included in the study. All patients were recruited from the PRAEGNANT network [50]. Patients had no prior exposure to any electronic assessment tools in the study in which they were currently included. If patients had prior contact with ePRO in other studies, they were not asked to participate (exclusion criteria).

After filling out their paper-based PRO questionnaires, the *nonexposed patients* were asked whether they would be interested and confident in using electronic assessments prospectively and whether there were any preexisting barriers. *Exposed patients* were provided with the actual electronic assessment application (ePROCOM). They were requested to fill out both the paper- and tablet-based PRO questionnaires so that the reliability of an ePRO tool could be analyzed. After filling out both questionnaires, they were also asked about their preferences toward future usage of either paper-based assessment or ePRO. The aim of ePROCOM was to evaluate the general patient acceptance and practicability of a Web-based application for a PRO-questionnaire for patients with aBC or mBC. The ePROCOM patients were asked to participate to compare the response behavior of patients in paper-based and Web-based questionnaires (publication in preparation). Inclusion criteria of ePROCOM were female gender, full legal age, aBC or mBC diagnosis, sufficient language skills in German, and signed declaration of consent. The ePROCOM patients were also asked to complete the questionnaire during an outpatient visit to the hospital under the supervision of an attending physician. We have previously reported on the influence of age, educational status, HRQoL, and technical skills of mBC patients [46].

The patients of both arms of the study (exposed and nonexposed) were informed about the aims of the study and were asked for their consent ex ante. The ethics committee gave prior consent for the study (project number 196/2015B02 and 089/2015B02). Randomization in this setting was not feasible, as patients were recruited from different studies. However, the trial design enabled identification of the main barriers in breast cancer patients for participating in ePRO in relation to the exposed versus nonexposed setting with regard to sociodemographic factors, therapy setting (aBC vs mBC), and HRQoL.

### Assessments

The assessment comprised 3 parts. The first part focused on the patientsʼ socioeconomic variables. The second part focused on HRQoL according to the EORTC QLQ‑C30, comprising 30 questions in 5 subscales, various symptom scales, and individual items related to the patientsʼ health status on a multidimensional level. We used only those 2 questions from the EORTC QLQ‑C30 that focused on the patient’s health status and HRQoL on a 7-point Likert scale (from 1=very poor to 7=excellent). The acceptance level and identification of barriers and acceptance, but not HRQoL, constituted the main focus of the analyses. Patients also completed the entire EORTC QLQ‑C30 questionnaire; data on every single function and symptom scale are available upon request. Mean values were calculated in accordance with the official EORTC guidelines, which require a separate score to be calculated for each scale. The scores ranged from 0 to 100 [51,52]. In the third part of the questionnaire, the patients were asked about preexisting technical skills such as use of electronic technology at home, routine usage of digital devices such as computers, Internet use, their general attitude toward electronic-based surveys, and potential barriers in relation to their health status. Furthermore, the patients in the nonexposed survey were also asked about existing technological support structures because they only completed the paper-based questionnaire, whereas the exposed group filled out both paper- and tablet-based questionnaires. The trial design was based on the Reach, Effectiveness, Adoption, Implementation, and Maintenance (RE-AIM) framework. This guidance plan was developed specifically for assessing the effectiveness of interventions and included aspects of reach, effectiveness, adoption, implementation, and maintenance [53,54].

### Statistical Analysis

**Figure 1:**
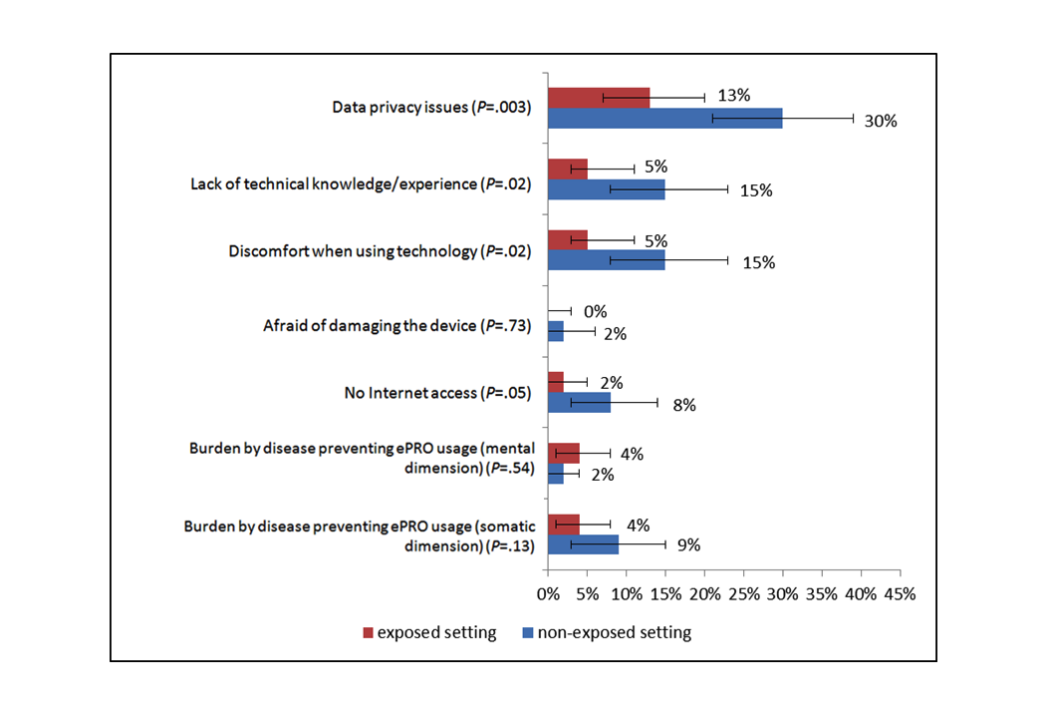
Barriers for using electronic-based patient-reported outcome.

A frequency analysis was first performed using the Statistical Package for the Social Sciences (SPSS) version 21 (IBM) to determine the descriptive characteristics of the collected data. The goal was to demonstrate how the barriers of technology-based surveys are distributed over the entire population. The influence of the barriers on the rejection of electronic-based surveys was also identified, and the barriers among patients with preferences for paper-based questionnaires and ePRO were compared in relation to socioeconomic variables, health status, and technical skills in self-perception. Differences between nonexposed and exposed patients were identified using chi-square tests (if the variables were dichotomous and binary coded) and Wilcoxon signed-rank tests in ordinal- and metric-scaled data, because the paired samples were not normally distributed in the Shapiro-Wilks test and in quantile-quantile plots. Furthermore, a relative risks analysis was calculated to identify the influence of ePRO exposure on usability and barriers. Subsequently, we performed univariate categorical regression (CATREG) analysis to ascertain regression context between ePRO exposure and willingness to use the identified barriers [55,56]. Mediation analyses (Hayes’ bias-corrected bootstrap) were then performed to expose the potential interferences of the regression model [56]. Finally, demand for technical support was measured through frequency analysis in the nonexposed group. Beforehand, we performed chi-square tests and Shapiro-Wilks test between mBC and aBC patients in both groups to identify possible statistically significant differences in relation to HRQoL and willingness to use. A bilateral *P* value of <.05 was considered statistically significant in all analyses (alpha=.05). The survey was conceived as an explorative study, in which all *P* values were to be understood purely descriptively and had no confirmatory value. Figure 1 was created in Microsoft Excel 2010.

## Results

### Sociodemographic Variables and Technical Skills

A total of 202 female breast cancer patients completed the PRO assessment (nonexposed group: n=96 patients; exposed group: n=106 patients). We did not find significant intragroup differences between aBC and mBC patients. Table 1 shows the sociodemographic characteristics of the study group. Nonexposed patients were significantly older compared with the exposed group, and their self-rated HRQoL was reported to be worse in the EORTC QLQ-C30 survey. However, the differences in HRQoL between both groups were not statistically significant. The level of education was significantly higher in the exposed group.

The technical skills are shown in Table 2. In all dimensions and at all levels, the self-reported technical skills were higher in exposed patients, including considerable time of computer and Internet use and higher frequency of tablet usage.

### Willingness to Use Technology-Based Surveys (ePRO)

The results for both treatment groups suggest that the introduction of electronic surveys will indeed improve clinical care and completion of ePRO questionnaires; however, there were significant differences between exposed and nonexposed patients. Exposed patients more often suggested that hospital care could be improved by using ePRO questionnaires and more frequently rated ePRO assessments as being more suitable, less tiring, and less difficult (Table 2). Before exposure to the ePRO application, both groups were asked about their potential ePRO assessment usage. Overall, the disposition for potential ePRO usage was high, with 77% of all patients indicating willingness. However, there were significant differences with regard to the HRQoL (Table 2). As the percentage of adjuvant patients was obviously higher in the exposed group, adjuvant patients showed higher usage willingness, whereas the nonexposed group (with a higher percentage of metastatic patients) showed less willingness. The ePRO willingness was 92% in exposed versus 59% in the nonexposed group.

Identifying existing barriers is crucial for future implementation of ePROs in routine clinical practice. The patients were asked whether there are any existing barriers related to privacy, technology, or disease that would negatively influence their willingness to use technology-based surveys. Multiple barriers in seven dimensions were identified, and most of them showed statistically significant differences between both groups in favor of the exposed patients (Figure 1). The most evident item was concern about data security, followed by two technological barriers (lack of technical knowledge; experience and discomfort when using technology). All barriers with statistically significant differences were reported more often in nonexposed patients. In contrast, differences in the burden of the disease as a reason for nonusage were not significant between both groups.

**Table 1:** Sociodemographic characteristics of exposed and nonexposed treatment groups. Statistically significant values presented in italics.

Sociodemographic variables		Exposed (n=106)	95% CI	Nonexposed (n=96)	95% CI	*P* value (alpha=.05)
**Age in years**						
	Mean (median)	51.0 (52)		56.68 (54)		*.001*
	Standard deviation [range (minimum-maximum)]	11.31 [54 (30-84)]		12.38 [60 (20-85)]		
**Level of education (1=lowest; 6=highest)**						
	Median	3.0		3.0		*.03*
	Interquartile range (25%-quartile-75%-quartile)	2.0 (3.0-5.0)		2.0 (2.0-4.0)		*.03*
	No qualification, n (%)	1 (.9)	(0.00-0.06)	1 (1)	(0.00-0.07)	.94
	Main/secondary school leaving certificate, n (%)	43 (40.6)	(0.32-0.50)	59 (61)	(0.53-0.69)	*.003*
	Advanced technical certificate, n (%)	19 (17.9)	(0.10-0.26)	15 (16)	(0.08-0.23)	.67
	High school diploma (“Abitur”), n (%)	33 (31.1)	(0.22-0.40)	13 (14)	(0.07-0.22)	*.003*
	Not specified, n (%)	10 (9.4)	(0.02-0.15)	8 (8)	(0.01-0.13)	.78
**Therapy setting**						
	Metastatic, n (%)	30 (28.3)	(0.19-0.35)	65 (68)	(0.62-0.76)	*.001*
	Adjuvant treatment, n (%)	76 (71.7)	(0.61-0.83)	31 (32)	(0.26-0.37)	*.001*
**Health-related quality of life (EORTC QLQ C-30)^a^**						
	Mean (median)	60.8 (66.67)	(0.55-0.66)	58.1 (58.3)	(0.52-0.63)	.45
	Standard deviation [range (minimum-maximum)]	23.75 [100 (0-100)]		21.0 [91.7 (0-91.7)]		.45

^a^EORTC QLQ‑C30: European Organization for Research and Treatment of Cancer Quality of Life Questionnaire-Core 30 item.

**Table 2:** Self-reported technical skills for metastatic and adjuvant patients. Statistically significant values presented in italics.

Technical skills and ePRO evaluation			Exposed		95% CI		Nonexposed		95% CI		*P* value (alpha=.05)	
**Computer skills (self-perception by the patients, 1=lowest; 4=highest)**												
	Median			3.0				2.0				*<.001*
	Interquartile range (25%-quartile-75%-quartile)			0.0 (3.0-3.0)				1.0 (2.0-3.0)				*<.001*
				**n=99**				**n=81**				
	Beginner/no skills (=1), n (%)			4 (4)		(0.01-0.08)		10 (12)		(0.06-0.18)		*.04*
	Basic (=2), n (%)			20 (20)		(0.12-0.28)		37 (46)		(0.37-0.58)		*<.001*
	Advanced (=3), n (%)			68 (69)		(0.58-0.77)		30 (37)		(0.27-0.47)		*<.001*
	Professional (=4), n (%)			7 (7)		(0.03-0.13)		4 (5)		(0.01-0.11)		.55
**Computer use, in years**												
	Mean (standard deviation)			17.49 (7.12)				16.73 (8.25)				.52
**Internet use, in years**												
	Mean (standard deviation)			13.57 (5.60)				11.84 (6.53)				.07
**Tablet PC use (1=lowest; 4=highest)**												
	Median			3.0				1.5				*<.001*
	Interquartile range (25%-quartile-75%-quartile)			3.0 (1.0-4.0)				2.0 (1.0-30)				*<.001*
				**n=94**				**n=66**				
	Not at all (=1), n (%)			33 (35)		(0.26-0.45)		33 (50)		(0.35-0.63)		.06
	A little (=2), n (%)			6 (6)		(0.02-0.12)		10 (15)		(0.08-0.23)		.07
	Moderate (=3), n (%)			13 (14)		(0.07-0.21)		19 (29)		(0.20-0.42)		*.02*
	Very much (=4), n (%)			42 (45)		(0.34-0.54)		4 (6)		(0.02-0.14)		*<.001*
				**n=104**				**n=86**				
Willingness to use technology-based surveys (ePRO), n (%)				96 (92.3)		(0.90-0.99)		51 (59)		(0.49-0.70)		*.001*
				**n=92**				**n=56**				
Do you think that the introduction of electronic surveys will improve clinical care?, n (%)				87 (95)		(0.89-0.99)		45 (80)		(0.70-0.89)		*.007*
**Comparison of e-based and paper-based questionnaires**												
	**ePRO is less suitable (=1), more suitable (=5)**											
		Median		4.0				3.0				*<.001*
		Interquartile range (25%-quartile-75%-quartile)		2.0 (3.0-5.0)				1.25 (3.0-4.25)				*<.001*
	**ePRO is more tiring (=1), less tiring (=5)**											
		Median		4.0				3.0				*<.001*
		Interquartile range (25%-quartile-75%-quartile)		2.0 (3.0-5.0)				1.0 (3.0-4.0)				*<.001*
	**ePRO is more difficult (=1), less difficult (=5)**											
		Median		4.0				3.0				*<.001*
		Interquartile range (25%-quartile-75%-quartile)		2.0 (3.0-5.0)				2.0 (2.0-4.0)				*<.001*

^a^ePRO: electronic-based patient-reported outcome.

**Table 3:** Relative risks of willingness to use and different barriers in exposed patients in relation to the nonexposed group. Statistically significant values presented in italics.

Willingness to use and barriers	Relative risk in exposed patients (95% CI)
Willingness to use ePRO^a^	*11.834* (4.405-31.794)
Data privacy issues	*0.371* (0.179-0.769)
Lack of technical knowledge/experience	0.372 (0.138-1.006)
Discomfort when using technology	*0.243* (0.77-0.761)
I am afraid of damaging the device	-
No Internet access	0.120 (0.15-0.976)
Burden of disease preventing ePRO usage (mental dimension)	0.363 (0.093-1.411)
Burden of disease preventing ePRO usage (somatic dimension)	2.089 (0.373-11.687)

^a^ePRO: electronic-based patient-reported outcome.

### Relative Risks, Regression, and Mediation Analyses

Table 3 shows the results of the probability analyses. It is apparent that the probability of willingness to use is almost 11 times higher after exposure in this collective, whereas the relative risks of existing barriers are obviously lower (especially data privacy issues and discomfort when using technology).

The CATREG analysis substantiates a statistically significant regression context between ePRO exposition and willingness, whereas the influence of the identified barriers was only low and partly not significant (Table 4) because the respective sample sizes of patients with existing barriers were too small for a valid calculation. Overall, 16.6% of the cases with expressed willingness to use can be attributed to exposure. Mediation effects of age and computer skills against the influence of exposure on willingness to use were only low (Table 5), whereas the mediation influence of education, HRQoL, and therapy setting were not statistically significant because the differences between exposed and nonexposed patients were too small. Including the variables of age and computer skills toward influence of exposure increased the explainability of the willingness to use aspect to 31.9%.

### Needs and Possible Technological Support Structures

After finding strongly distinct barriers for ePRO among nonexposed patients, we asked them how they would rate the importance of 5 possible support services to help them complete a Web-based questionnaire about medical treatment, side effects, health status, and HRQoL (Table 6). On-site support services were rated as being moderately or highly important by 38%. A total of 32% patients expressed desire for a technical briefing for relatives who would support them while using the ePRO tool. Technical telephone support was rated as moderately important or very important by 52% of the nonexposed patients. The most relevant topic was data security, and 71% of the patients wanted to have full information regarding data protection measures (moderate and high importance). At least 61% would appreciate receiving direct feedback after using the ePRO application.

**Table 4:** Categorical regression analyses. Statistically significant values presented in italics.

Influence of exposure	*R*	*R*^2^	Beta	*P* value (alpha=.05)
Willingness to use ePRO^a^	.407	.166	.407	*<.001*
Data privacy issues	.207	.043	−.207	*.004*
Lack of technical knowledge/experience	.129	.017	−.129	*<.001*
Discomfort when using technology	.166	.028	−.166	*.01*
I am afraid of damaging the device	.052	.003	−.052	.43
No Internet access	.138	.019	−.138	*.02*
Burden of disease preventing ePRO usage (mental dimension)	.106	.011	−.106	.12
Burden of disease preventing ePRO usage (somatic dimension)	.045	.002	−.045	.52

^a^ePRO: electronic-based patient-reported outcome.

**Table 5:** Willingness to use: mediation effect of sociodemographics, skills, and health-related quality of life.

Willingness to use: mediation effect of variables	*R*_Mod_	*R*^2^_Mod_	*P* value (alpha=.05)	Indirect effect of X^a^ on Y^b^	95% CI
Age	.246	.062	*<.001*	.363	(0.073-0.867)
Level of education	.152	.023	*.04*	.235	(0.017-0.608)
Computer skills	.302	.091	<.001	.536	(0.196-1.976)
Health-related quality of life	.085	.007	.28	.057	(−0.035 to 0.353)
Therapy setting	.092	.008	.18	.63	(−0.042 to 0.157)
R^2^_ges_ = R^2^ + R^2^_Mod / Age_ + R^2^_Mod / Skills_ = .166 + .062 + .091 = .319					

^a^X=exposure/no exposure.

^b^Y=willingness to use.

**Table 6:** Electronic-based patient-reported outcome preferences regarding technical support structures: How important would you rate the following support services to complete an electronic-based patient-reported outcome questionnaire during the hospital visit about your medical (after) treatment, your side effects, your health status, and your quality of life?

Support variables		Nonexposed setting	
		n (%)	95% CI
**Technical briefing and onboarding completed on site, (N=69)**			
	Not at all	26 (38)	(0.28-0.53)
	A little	16 (23)	(0.15-0.37)
	Moderate	13 (19)	(0.07-0.23)
	Very much	14 (20)	(0.10-0.32)
**Technical briefing should include relatives, (N=64)**			
	Not at all	35 (55)	(0.42-0.68)
	A little	9 (14)	(0.07-0.25)
	Moderate	8 (13)	(0.05-0.22)
	Very much	12 (19)	(0.08-0.27)
**Telephone support, (N=64)**			
	Not at all	17 (27)	(0.18-0.40)
	A little	14 (22)	(0.12-0.32)
	Moderate	12 (22)	(0.12-0.32)
	Very much	19 (30)	(0.18-0.42)
**Transparency of data privacy, (N=70)**			
	Not at all	11 (16)	(0.10-0.28)
	A little	9 (13)	(0.06-0.24)
	Moderate	12 (17)	(0.08-0.27)
	Very much	38 (54)	(0.38-0.63)
**I get a direct feedback (from a doctor or the hospital), (N=68)**			
	Not at all	17 (25)	(0.18-0.40)
	A little	10 (15)	(0.07-0.27)
	Moderate	14 (21)	(0.10-0.28)
	Very much	27 (40)	(0.25-0.50)

## Discussion

### Principal Findings

The majority of breast cancer patients expressed interest in adopting ePRO based on the impression that ePRO would positively impact hospital care and based on enhanced usability (more suitable, less tiring, and less difficult to read than paper-based PRO). Differences in relation to the setting of the survey and the patient’s self-reported health status were significant because the HRQoL was higher and the number of metastatic patients was lower in the exposed group. Patients in the nonexposed group more often had reservations and were critical toward ePRO, and their willingness to use corresponding tools was because of the following barriers: Patients were often afraid of using technical devices such as tablet PCs, (especially those with metastatic diseases in the nonexposed group), and they were concerned about data privacy issues and disease-related barriers (Figure 1). Thus, the willingness to participate in ePRO assessments can be increased by offering an ePRO tool, and the influence of barriers can also be reduced in metastatic patients. Our data demonstrated that patients generally had prevalent reservations regarding electronic assessment before exposure; however, they showed willingness to use electronic assessments after exposure. Whereas 16.6% of the cases expressing willingness to use were attributed to exposure (Table 4), mediation effects of age and computer skills against exposure’s influence were only low (Table 5). We found higher barriers in the nonexposed group characterized by lower HRQoL and a higher number of metastatic patients (Table 1), which suggests that health status influences the acceptance of ePRO and the emergence of barriers. The dimensions of reach and effectiveness of the RE-AIM framework could be analyzed for future improvements. The development of ideal ePRO tools has to consider the identified barriers (technical skills, HRQoL, and sociodemographic aspects) for utilization of ePRO, preferably in the general patient population and independent of their multidimensional characteristics.

### Comparison With Prior Work

The results of this study contrast with those of a previous study, which identified no differences in the feasibility assessment of ePRO in relation to HRQoL [38]. The number of ePRO systems has increased in recent years, especially in oncology clinical practice, but other studies did not focus on the possible barriers to usability [57,58]. We have not found any studies in which cancer patients were asked about their barriers. Our group previously showed that older mBC patients (>62 years) with higher burden of disease may be less willing to complete ePRO questionnaires [46]. In this study, some significant barriers in relation to HRQoL, survey setting, and cancer-related restrictions were identified, whereas other reports only described the acceptance of ePRO without ascertaining barriers [40-42,57,58]. Our results agree with Basch et al [38], who reported higher self-reported computer experience (and thus potentially higher acceptance for ePRO) in patients with higher HRQoL. No other studies identified specific barriers related to technical skills, HRQoL, and sociodemographic issues as predictive factors for nonresponse in ePRO.

### Limitations and Relevance

Our study was developed as a bicentric trial, and the patients were surveyed while they were receiving chemotherapy intervention. We did not enquire about the tumor stage, extent of metastasis, and the administered therapy. Furthermore, psycho-oncological information was not gathered, although psycho-oncological distress is a commonly associated burden that could potentially influence the willingness to use ePRO. There was no significant mediation effect of the therapy setting (aBC and mBC), although the number of metastatic patients was significantly higher in the nonexposed group. Also, HRQoL seemed not to be an influencing factor for willingness to use, as there were no significant differences in relation to HRQoL between exposed and nonexposed patients and no significant mediation effect. As it is known that low HRQoL and metastatic situation influence the willingness to use [46], willingness was assumed to be poor in mBC patients at the beginning, because metastasis was associated with poorer HRQoL. Probably, there were no differences in HRQoL (both in comparison of exposed and nonexposed patients as well as in the intragroup analyses), but this hypothesis could not be confirmed in this study. Hence, it can be postulated that a metastasis situation has a negative effect on usability compared with patients in adjuvant therapy especially if it results in a poorer HRQoL. An indirect effect was shown by the fact that for the exposed group significantly less metastasized patients could be recruited. The aspects of age and computer skills appeared as significant limitations, as exposed patients were significantly younger and had significantly better skills, which indicates that especially younger patients with previous experience in technology could be motivated to use ePRO.

The most important result of the study was the fact that the survey setting (nonexposed vs exposed setting) could influence the willingness to use ePRO and the probability of barriers in all mBC and aBC breast cancer patients. The willingness among exposed patients was higher, as only the patients who could envisage answering HRQoL questions with a tablet could be included in this study. In total, 130 patients declined to participate in this group, so the total impact might be negligible; however, this limitation generally occurs in other ePRO trials. Because patients with barriers were rather unwilling to take part in the study, it is unclear how exposed patients are influenced by the approach of the study personnel to participate. Therefore, the barriers in the nonexposed group must be taken seriously because they could also represent patients with potential reservations about ePRO. The comparison between nonexposed and exposed patients shows that the willingness among women with breast cancer can be increased, and barriers can be reduced by educating the patients. To prevent statistical bias in future surveys and to increase the reliability of ePRO questionnaires, the identified barriers must be eliminated. Patients with cancer, who are often limited by their disease, should be thoroughly informed about privacy security issues and the universal handling of such confidential information to address their concerns and increase their potential willingness to use ePRO applications.

### Conclusions

Although general patient acceptance of ePRO was high, we identified technical and disease-related barriers. These findings underscore the need to be aware of such barriers and to eliminate them to enhance the practicability of ePRO and ensure data accuracy, reliability, and validity for future ePRO assessments to measure HRQoL. Whereas fewer preexisting barriers were found in younger breast cancer patients, older patients with poorer HRQoL and less preexisting technical skills more frequently reported barriers for ePRO. Our study showed that barriers can be overcome after exposure and the willingness to participate in ePRO assessments significantly increased. Hence, the dimensions of reach and effectiveness of the RE-AIM framework, in particular, were analyzed in this paper. The development of ideal ePRO tools has to consider the identified barriers (technical skills, HRQoL, and sociodemographic aspects) for the utilization of ePRO, preferably in the general patient population and independent of their multidimensional characteristics. Tailored educational and support services need to be implemented and evaluated in future research to relieve reservations and increase ePRO compliance. Willingness to use ePRO is dependent on sociodemographic aspects, technical skills, HRQoL, and therapy setting, but patients’ acceptance of the tool can be increased when they experience it firsthand.

## Abbreviations

aBC: adjuvant breast cancer
AEs: adverse events
CATREG: categorical regression
eHealth: electronic health
EORTC QLQ-C30: European Organization for Research and Treatment of Cancer Quality of Life Questionnaire-Core 30 item
ePRO: electronic-based patient-reported outcome
FACT-G: Functional Assessment of Cancer Therapy-General
HRQoL: health-related quality of life
mBC: metastatic breast cancer
PRO: patient-reported outcome
PROCOM: Patient-Reported Outcomes and Compliance Analysis
RE-AIM: Reach, Effectiveness, Adoption, Implementation, and Maintenance
SPSS: Statistical Package for the Social Sciences
